# Nitrogen availability and plant–plant interactions drive leaf silicon concentration in wheat genotypes

**DOI:** 10.1111/1365-2435.14170

**Published:** 2022-09-01

**Authors:** Felix de Tombeur, Taïna Lemoine, Cyrille Violle, Hélène Fréville, Sarah J. Thorne, Sue E. Hartley, Hans Lambers, Florian Fort

**Affiliations:** ^1^ CEFE, Univ Montpellier, CNRS, EPHE, IRD Montpellier France; ^2^ School of Biological Sciences and Institute of Agriculture The University of Western Australia Perth WA Australia; ^3^ AGAP, Univ Montpellier, CIRAD, INRAE, Institut Agro Montpellier France; ^4^ Department of Biology University of York York UK; ^5^ School of Biosciences University of Sheffield Sheffield UK; ^6^ CEFE, Univ. Montpellier, L'Institut agro, CNRS, EPHE, IRD Montpellier France

**Keywords:** agroecology, facilitation, genotype mixture, intraspecific variation, nutrient limitation, phenotypic plasticity, plant competition, plant height

## Abstract

1. Estimating plasticity of leaf silicon (Si) in response to abiotic and biotic factors underpins our comprehension of plant defences and stress resistance in natural and agroecosystems. However, how nitrogen (N) addition and intraspecific plant–plant interactions affect Si concentration remains unclear.
2. We grew 19 durum wheat genotypes (*Triticum turgidum* ssp. *durum*) in pots, either alone or in intra‐ or intergenotypic cultures of two individuals, and with or without N. Above‐ground biomass, plant height and leaf [Si] were quantified at the beginning of the flowering stage.
3. Nitrogen addition decreased leaf [Si] for most genotypes, proportionally to the biomass increase. Si plasticity to plant–plant interactions varied significantly among genotypes, with both increases and decreases in leaf [Si] when mixed with a neighbour, regardless of the mixture type (intra‐/intergenotype). Besides, increased leaf [Si] in response to plant–plant interactions was associated with increased plant height.
4. Our results suggest the occurrence of both facilitation and competition for Si uptake from the rhizosphere in wheat mixtures. Future research should identify which leaf and root traits characterise facilitating neighbours for Si acquisition. We also show that Si could be involved in height gain in response to intraspecific competition, possibly for increasing light capture. This important finding opens up new research directions on Si and plant–plant interactions in both natural ecosystems and agroecosystems. More generally, our results stress the need to explore leaf Si plasticity in responses to both abiotic and biotic factors to understand plant stress resistance.

Read the free Plain Language Summary for this article on the Journal blog.

## Graphical abstract

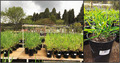

## 1 INTRODUCTION

Silicon (Si), taken up from soil as monosilicic acid and deposited in plant tissues as silica (SiO_2_·nH_2_O), increases plant resistance to a wide range of biotic and abiotic stresses (e.g. water stress, metal toxicity, pathogens and herbivory) (Cooke & Leishman, 2016; Debona et al., 2017; Hartley & DeGabriel, 2016; Massey & Hartley, 2006) and confers mechanical strength to plants (Epstein, 1994; Raven, 1983). The essentiality of Si for plants remains challenging to assess (Coskun et al., 2019; Epstein, 1994) but increased resistance to herbivores and stress alleviation following Si fertilisation can lead to increased plant primary productivity and crop yields (Liang et al., 2015; Savant et al., 1999; Tubana et al., 2016; Xu et al., 2020). Because graminoid crop species can exhibit very high Si concentrations ([Si]) (e.g. up to 20% of SiO_2_ in rice; Klotzbücher et al., 2018), the beneficial role of Si in agriculture is well recognised, and Si is routinely applied to croplands in many countries (e.g. China, Japan, USA, Brazil) (Datnoff et al., 2001; Yan et al., 2018). Thus, it is important to understand the factors affecting plant Si nutrition but, to date, we still have limited knowledge of how soil nutrient availability and interactions between plants affect Si concentration.

Increasing evidence suggests that plant Si concentration depends on soil nutrient status (de Tombeur, Laliberté, et al., 2021; Johnson et al., 2021; Minden et al., 2021; Quigley et al., 2020). In particular, decreases in Si concentration and resulting Si‐based defences following nitrogen (N) fertilisation have recently been reported for different grassland/pasture species (Johnson et al., 2021; Minden et al., 2021; Quigley et al., 2020) (but see Moise et al., 2019). This has been attributed to the investment in ‘cheap’ Si versus relatively ‘more expensive’ carbon (C) (Raven, 1983) during N stress (Johnson et al., 2021; Minden et al., 2021) and reflects trade‐offs between plant growth rate and carbon‐ or Si‐based defences within *Poaceae* family (Massey et al., 2007). However, past studies have generally focused only on a single, non‐cultivated genotype (Johnson et al., 2021; Minden et al., 2021). Significant genotypic variation in Si concentration has been reported in rice and wheat (Ma et al., 2007; Merah et al., 1999; Talukdar et al., 2019), so the plasticity (i.e. production of multiple phenotypes from a single genotype depending on environmental conditions; Miner et al., 2005) of leaf [Si] in response to N fertilisation might differ among genotypes, but this has not yet been tested.

So far, the influence of plant–plant interactions on plant Si nutrition has received surprisingly little attention in the literature (but see Garbuzov et al., 2011; Ning et al., 2017, 2021), especially compared with other nutrients (Li et al., 2014). At the interspecific level, Ning et al. (2021) showed that rice accumulates significantly more Si when grown with water spinach (*Ipomoea aquatica* Forsk)—a low Si‐accumulating species—compared with a rice monoculture, possibly through the effect of root exudates on soil Si mobilisation (de Tombeur, Cornelis, et al., 2021; Ning et al., 2021). However, when two grasses with high Si‐concentration (*Poa annua* and *Lolium perenne*) were investigated, such interspecific facilitation on Si concentration was not observed (Garbuzov et al., 2011). The influence of plant–plant interactions on Si concentration at the intraspecific level, to our knowledge, has received no attention, either in intra‐genotypic cultures or intergenotypic mixtures. It is important to consider both intra‐ and intergenotypic cultures because facilitation for Si uptake in the rhizosphere might prevail over competition when genotypes are functionally different (e.g. they contrast in nutrient‐acquisition strategies and/or Si demand). Furthermore, both types of genotypic cultures should be considered because intragenotypic stands are typical of modern agriculture, but there is increasing interest in the role of genetic diversity in increasing the sustainability of agriculture as greater intraspecific diversity may increase productivity and resistance to pests and pathogens (Barot et al., 2017; Hajjar et al., 2008; Litrico & Violle, 2015; Montazeaud et al., 2022).

Finally, leaf Si has been linked to different plant architecture traits that could in turn influence competition for light capture, including decreasing leaf insertion angle and leaf arc/straightness (Ando et al., 2002; de Tombeur, Cooke, et al., 2021; Yamamoto et al., 2012; Zanão Júnior et al., 2010), and increasing plant height (Gong et al., 2003; Ma et al., 1989; Zanão Júnior et al., 2010). As such, we might expect some relationships between the Si concentration of a genotype and the outcomes of plant–plant interactions (i.e. in this case, biomass loss or gain when mixed with a neighbour). It remains challenging to predict potential links between Si and competition outcomes, since greater plant height might increase competition intensity (Falster & Westoby, 2003; Violle et al., 2009), but decreasing leaf insertion angle and arc reduces the light extinction coefficient inside the canopy and may thus decrease competition intensity (Ando et al., 2002). Nevertheless, studies on Si benefits against biotic and abiotic stresses have greatly expanded during the last 10 years (Coskun et al., 2019), and investigating previously overlooked functions of silicification, such as its influence on plant architecture and potential impact on plant–plant interactions, is thus needed.

Here, we studied 19 genotypes of durum wheat (*Triticum turgidum* ssp. *durum*), a major staple crop, which we grew in pots, either alone, in intragenotypic culture or in intergenotypic culture, at two levels of N availability. We quantified plant above‐ground biomass, plant height and leaf [Si] to (a) evaluate intraspecific variation in leaf [Si] among the 19 genotypes, (b) estimate plasticity of leaf [Si] in response to N fertilisation and plant–plant interactions and (c) explore potential relations between leaf [Si] and competition outcomes. The variation of leaf [Si] among genotypes, as well as plasticity in leaf [Si] in response to N fertilisation, was tested on genotypes grown alone to avoid a neighbour effect. How plant–plant interactions affect leaf [Si], either in intra‐ or intergenotypic cultures and with or without N addition, was tested by comparing the leaf [Si] of plants alone with that of plants in interaction. Finally, we tested correlations between genotype leaf [Si] and their response to competition in terms of biomass/height losses/gains to explore potential links between [Si] and competition outcomes. We hypothesised a decrease in leaf [Si] following N fertilisation. We further hypothesised that wheat genotypes would vary in both their Si concentrations, and in their response to N fertilisation and plant–plant interactions.

## 2 MATERIALS AND METHODS

### 2.1 Experimental design

We selected 19 durum wheat genotypes [*T. turgidum* ssp. *Durum* (Desf.)] from the Evolutionary Pre‐breeding pOpulation (EPO), a population of 180 genotypes with high phenotypic and genotypic diversities (David et al., 2014). The 19 genotypes represented a large phenotypic diversity on below‐ and above‐ground traits. The 19 genotypes were grown either alone in *single* (alone in the pot), in *intragenotypic culture* (two plants of the same genotype in the same pot) or in *intergenotypic culture* (two plants from different genotypes in the same pot), hereafter *growth modalities*, with two levels of N (treatment N^+^ and N^−^), and in triplicate. We randomly assembled 26 *intergenotypic* mixtures among the 171 possibilities. The modality *single* thus represents 114 individuals (19 genotypes × 2 N levels × 3 replicates), *intragenotypic culture* 228 individuals (2 plants × 19 genotypes × 2 N levels × 3 replicates) and *intergenotypic culture* 312 individuals (26 mixtures of 2 plants × 2 N levels × 3 replicates). In total, 384 pots and 654 wheat individuals were considered.

### 2.2 Growth conditions

The experiment was conducted at the CEFE experimental field (Montpellier, France) from January to May 2021, in outdoor conditions. We used a randomised complete block design using three blocks (one replicate in each block). Plants were grown in 4‐L plastic pots (18.5 cm diameter; 21.5 cm depth) filled with approximately 4.5 kg of local soil (52% sand, 27% silt and 21% clay; 6.9% CaCO_3_; 4.1% organic carbon; 0.21% total N; pH 8.0), and amended with PK fertiliser (0.38 g per pot; P_2_O_5_ and K_2_O). The effect of plant–plant interactions on plant Si uptake might be influenced by soil Si availability (Ning et al., 2021). Here, although not quantified, we expect Si availability to be rather high in this young, high‐pH and clay + silt‐rich soil (Cornelis & Delvaux, 2016). Indeed, a recent analysis of soil Si availability in French soils shows that this soil type exhibits the highest Si concentrations extracted with CaCl_2_ and is unlikely to be Si limited (Caubet et al., 2020). Two seeds per plant were sown in each pot and the largest plant was kept after germination. Pots of the N^+^ treatment received N four times during the experiment, for a total input of 0.94 g N per pot, whereas pots of the N^−^ treatment did not receive any N fertilisation. Plants were not protected from the rain and were watered with amounts to avoid water excess or deficit.

### 2.3 Plant height, biomass and leaf [Si] measurements

Vegetative plant height, plant above‐ground biomass and leaf [Si] were quantified at the beginning of the flowering stage. Vegetative height was measured as the distance between the soil surface and the tallest leaf without stretching the plant leaf. The leaf [Si] was quantified with an X‐ray fluorescence spectrometer (Reidinger et al., 2012). Briefly, three most recent ligulate adult leaves were sampled on each individual, dried at 60°C for 72 h and ball‐milled (Retsch MM400 Mixer mill) for 3 min at a frequency of 20 Hz. Ground samples were pressed at 10 tons into pellets using a manual hydraulic press (Specac). Si analyses were performed using a Nitron XL3t900 GOLDD XRF analyser (Thermo Scientific). Silicon‐spiked synthetic cellulose was used for calibration, and analyses were performed under helium atmosphere to avoid signal loss by air absorption (Reidinger et al., 2012). A reading was taken of each side of the pellet, approximately 1 h apart, to account for *u*‐drift in the instrument (Johnson, 2014). The concentration of Si in these three most recent ligulate adult leaves (in % of Si by dry weight) was considered to capture the intraspecific variation in leaf [Si] among the genotypes, the response to N fertilisation and plant–plant interactions, and potential relations between leaf [Si] and competition outcomes. Finally, all plant materials were harvested, dried at 60°C for 72 h and weighed to obtain above‐ground biomass.

### 2.4 Statistical analyses

#### 2.4.1 Variation in leaf [Si] among genotypes and response to N fertilisation

Variation in leaf [Si] among the 19 wheat genotypes and their plasticity to N fertilisation were assessed only for the *single* plants to discriminate it from the neighbour effect. For both N treatments, differences in leaf Si across the 19 genotypes were tested by a one‐way analysis of variance (ANOVA). To quantify the plasticity of leaf [Si] in response to N fertilisation among the 19 genotypes, we calculated log response‐ratios (hereafter logRR) as the logarithm of ratios between individual trait values and corresponding genotype‐mean values in N^−^, as follows:

$$\text{logRR}=\log10\left(\frac{\text{leaf}{\left[\mathrm{Si}\right]}_{N^{+}}}{\bar{\text{leaf}{\left[\mathrm{Si}\right]}_{N^{-}}}}\right).$$

Differences in logRR among genotypes were tested by ANOVA, and genotype‐mean logRR significantly different from zero were assessed with Student's *t*‐tests. A logRR below zero means that the treatment significantly decreased the trait values, while the opposite is true for logRR above zero.

#### 2.4.2 Plasticity to plant–plant interactions

We first tested differences in leaf [Si] among the treatments *single*, *intra‐* and *intergenotypic cultures* by ANOVA followed by post hoc tests using the ‘multcomp’ package (Hothorn et al., 2008) for both N treatments. To quantify the plasticity of leaf [Si] to plant–plant interactions, we calculated logRR as the logarithm of ratios between individual trait values and corresponding genotype‐mean values in *single*, independently for both N treatments. *Intra‐* and *intergenotypic culture* treatments were considered either separately or pooled together as a global factor ‘plant–plant interactions’ to contrast single versus two‐plant cultures in the analyses. Spearman rank correlation coefficients were calculated to test whether the ranking in genotype‐mean logRR were conserved between both N treatments and between *intra‐* and *intergenotypic cultures*. For the *intergenotypic culture* treatment, we further tested if neighbour identity influenced leaf [Si] by ANOVA, and for both N treatments.

#### 2.4.3 Relationships between leaf [Si], plant height and biomass

We first tested differences in plant above‐ground biomass and plant height across the different treatments (N and growth modality) by ANOVA, followed by post hoc tests. Relationships between above‐ground biomass/plant height (dependent variables) and leaf [Si] (independent variable) were then tested through mixed‐effect models with genotype identity as a random factor, using the package ‘nlme’ (Pinheiro et al., 2022). Models involving only the *single* individuals included both N treatments to test if a N‐induced decrease in biomass affects leaf [Si], while models considering only plants with a neighbour were run separately for each N treatment.

To test whether high‐Si genotypes lost more or gain more biomass as a response to plant–plant interactions, we tested the significance of relationships between the logRR of plant biomass in response to plant–plant interactions and genotype‐mean leaf [Si] in *single* by regression analyses, and for both N treatments.

For each model, residuals were inspected visually to check assumptions. Appropriate variance structures were specified in a second model if required (Zuur et al., 2009). All analyses were conducted in the R environment (R Core Team, 2021).

## 3 RESULTS

### 3.1 Intraspecific variation in leaf [Si] and plasticity to N fertilisation

**FIGURE 1:**
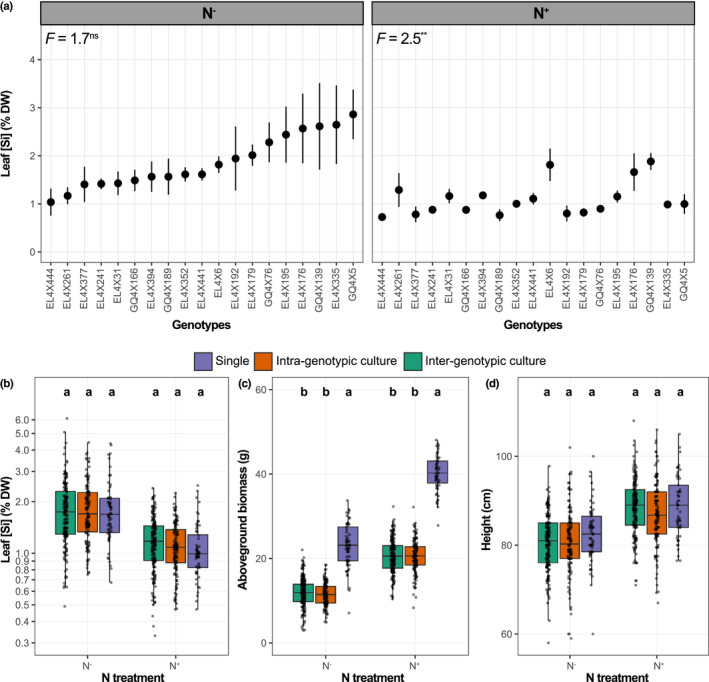
Leaf silicon concentrations ([Si]) of 19 durum wheat genotypes grown alone (*single*) and for two levels of N availability (means ± *SE*; *n* = 3) in (a). Boxplots showing the effects of plant growth modalities (*single*, *intra‐* and *intergenotypic culture*) on leaf [Si] in (b), plant above‐ground biomass in (c) and plant height in (d), for each N treatment. In (a), data are ranked by increasing genotype‐mean leaf [Si] in the N^−^ treatment for both plots, and results of ANOVA (*F*‐values) conducted between the genotypes are given. In (b)–(d), the central horizontal bar in each box shows the median, the box represents the interquartile range (IQR) and the whiskers show the location of the most extreme data points that are still within a factor of 1.5 of the upper or lower quartiles. Each point indicates one individual, and the *y*‐axis for leaf [Si] in (b) is on a logarithmic scale to improve visualisation. Different letters indicate significant differences (*p* < 0.05) between *single*, *intra‐* and *intergenotypic culture* within an N treatment. ***p < 0.001; **p < 0.01; *p < 0.05; ns, not significant.

**FIGURE 2:**
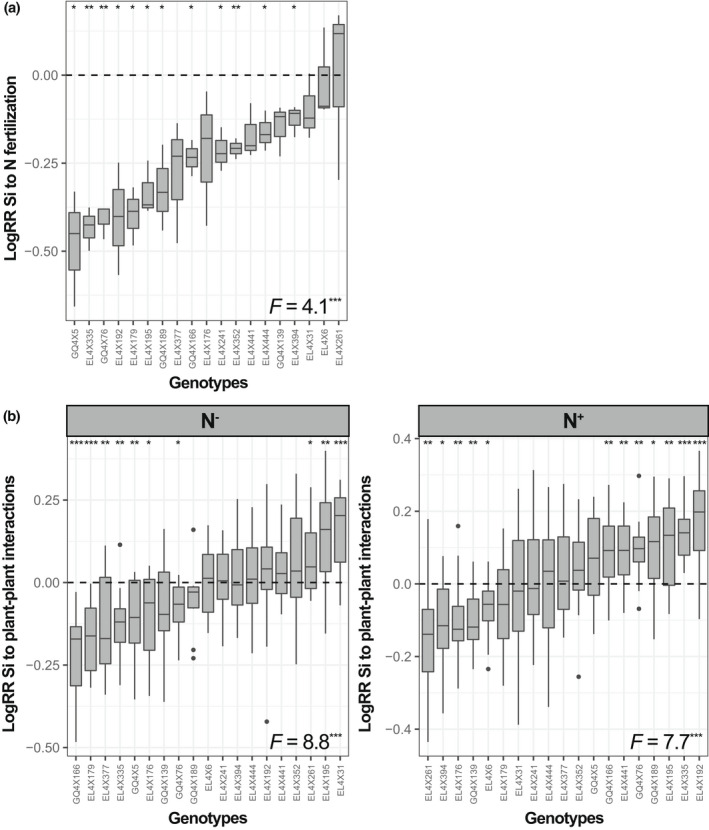
Variation in log response ratios (logRR) of leaf silicon (Si) concentrations to nitrogen (N) fertilisation for the *single* plants in (a) and to plant–plant interactions for both N treatments in (b) among 19 wheat genotypes. Both *intra‐* and *intergenotypic culture* were considered together in the analysis in (b) (see Figure S1 for separate analyses). Data are ranked by increasing genotype‐mean logRR. The central horizontal bar in each box shows the median, the box represents the interquartile range (IQR), the whiskers show the location of the most extreme data points that are still within a factor of 1.5 of the upper or lower quartiles, and black points are values that fall outside the whiskers. Results of ANOVA (*F*‐values) conducted between the genotypes are given. LogRR significantly different from zero following student *t‐*tests are indicated with stars. ***p < 0.001; **p < 0.01; *p < 0.05; ns, not significant.

Without N fertilisation, genotype‐mean leaf [Si] ranged from 1.0% to 2.9%, but did not significantly differ among genotypes (*p* = 0.09, Figure 1a). N fertilisation resulted in an overall decrease in leaf [Si] of 42%, with genotype‐mean ranging from 0.7% to 1.9% and that differed significantly among genotypes (Figure 1a). The response of leaf [Si] to N fertilisation (logRR) varied significantly among genotypes, and N fertilisation significantly decreased leaf [Si] for 12 out of the 19 genotypes (logRR < 0) (Figure 2a).

### 3.2 Plasticity to plant–plant interactions

We found no overall effect of growth modality (*single*, *intra‐* or *intergenotypic culture*) on leaf [Si], whether plants were N‐fertilised or not (Figure 1b). However, plasticity in leaf [Si] in response to plant–plant interactions (logRR) varied significantly among genotypes for both N treatments (Figure 2b). The presence of a neighbour significantly decreased leaf [Si] for seven genotypes in the N^−^ and for five genotypes in the N^+^ treatments (logRR < 0), and significantly increased leaf [Si] for three genotypes in the N^−^ and for seven genotypes in the N^+^ treatments (logRR > 0) (Figure 2b).

**FIGURE 3:**
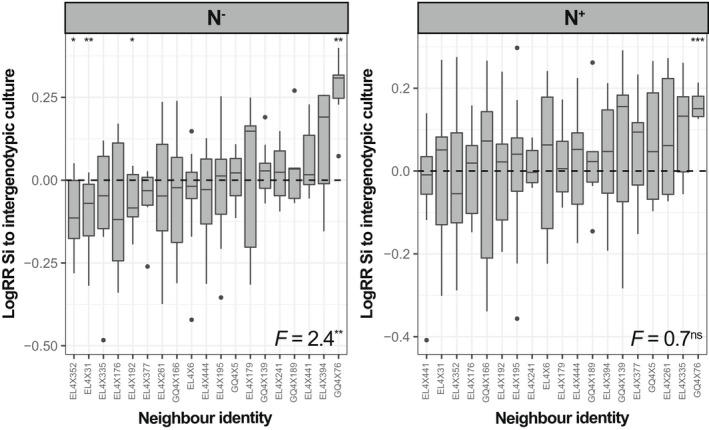
Variation in log response ratios (logRR) of leaf silicon (Si) concentrations to *intergenotypic culture* for both N treatments as a function of neighbour identity. Data are ranked by increasing neighbour identity‐mean logRR for both plots. The central horizontal bar in each box shows the median, the box represents the interquartile range (IQR), the whiskers show the location of the most extreme data points that are still within a factor of 1.5 of the upper or lower quartiles, and black points are values that fall outside the whiskers. Results of ANOVA (*F*‐values) conducted between the neighbour identity are given. LogRR significantly different from zero following student *t‐*tests are indicated with stars for the N^−^ treatment. ***p < 0.001; **p < 0.01; *p < 0.05; ns, not significant.

Genotypes varied significantly in their responses to plant–plant interactions also within the *intra‐* and *intergenotypic culture* treatments and for both N treatments (see Figure S1). Genotype‐mean responses were not consistent between N treatments (Spearman's coefficient *ρ* = −0.06 and *p* = 0.80 for intragenotypic culture; *ρ* = −0.12 and *p* = 0.64 for intergenotypic culture) but were consistent between the *inter‐* and *intragenotypic culture* treatments (*ρ* = 0.75 and *p* < 0.001 for N^−^; *ρ* = 0.73 and *p* < 0.001 for N^+^). Despite this, in the *intergenotype culture* treatment, the responses of leaf [Si] to plant–plant interactions significantly varied with neighbour identity in N^−^ (Figure 3). In particular, leaf [Si] responses were significantly below 0 for three neighbours and above 0 for one neighbour. Interestingly, this latter neighbour (GQ4X76) had the highest positive effect of leaf [Si] also in N^+^ (Figure 3).

### 3.3 Plant height, above‐ground biomass and responses to N fertilisation and plant–plant interactions

Overall, N fertilisation increased plant biomass and height, while the presence of a neighbour decreased biomass for both N treatments but had no significant effect on plant height (Figure 1c,d).

**FIGURE 4:**
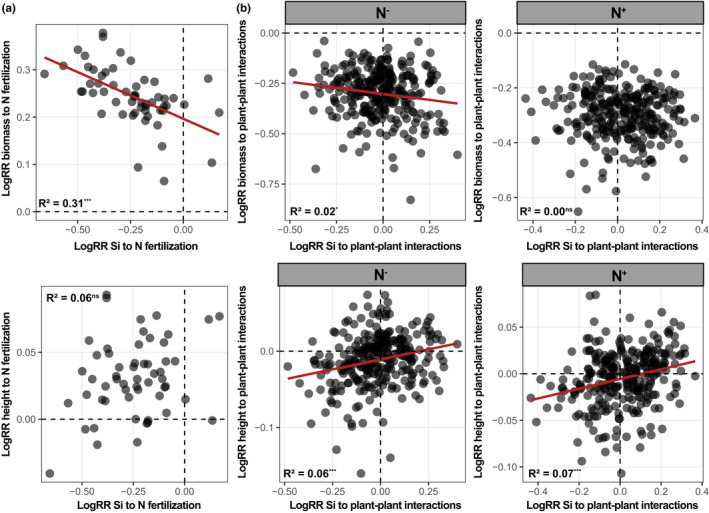
Relationships between the log response ratio (logRR) of leaf silicon (Si) concentrations and those of biomass and height to nitrogen (N) fertilisation for the *single* in (a) and to plant–plant interactions for both N treatments in (b). Both *intra‐* and *intergenotypic culture* were considered together as ‘plant–plant interactions’ in the analyses (see Figure S3 for *s*eparate analyses). Red lines indicate regression lines between variables, and multiple *R*‐squared are given. ***p < 0.001; **p < 0.01; *p < 0.05; ns, not significant.

We found a strong significant negative relationship between leaf [Si] and above‐ground biomass, but not plant height, for *single* plants (model including both N treatments) (Table 1). We found a strong negative relationship between the plasticity (logRR) in leaf [Si] and that of biomass in response to N fertilisation (Figure 4a), suggesting that larger increase in plant biomass following N fertilisation implied a stronger decrease in leaf [Si], and confirming the strong dependency between these two traits when N was manipulated. In contrast, plasticity of plant height to N fertilisation was not related to that of leaf [Si] (Figure 4a).

**TABLE 1:** Results of the mixed‐effect models (genotype as random factor) testing the effects of leaf silicon (Si) concentrations on above‐ground plant biomass and height for *single* plants (both nitrogen (N) levels in the analyses), and for plants in interactions for both N levels separately (*intra*‐ and *intergenotypic* treatments combined; see Table S1 for separated analyses)

	Single			Inter‐ and intragenotypic culture					
	N^−^ and N^+^ combined			N^−^			N^+^		
	Slope	*F*‐value	*p*‐value	Slope	*F*‐value	*p*‐value	Slope	*F*‐value	*p*‐value
Biomass ~ Leaf Si	−8.4	74.6	<0.001	−0.6	4.8	<0.05	−0.5	0.6	0.43
Height ~ Leaf Si	−1.6	4.2	<0.05	1.8	11.1	<0.001	5.6	31.7	<0.001

In the models considering plants with a neighbour, we also observed a slight negative relationship between leaf [Si] and above‐ground biomass, but only in the N^−^ treatment (Table 1). When *intra‐* and *intergenotypic culture* were considered separately, none of the biomass‐Si relationships were significant (Table S1). However, significant positive relationships between plant height and leaf [Si] were identified for both N treatments (Table 1), and within the *intra‐* and *intergenotypic culture* (Table S1).

Plasticity in plant biomass and plasticity in height to the presence of a neighbour were significantly related to genotype‐mean leaf [Si] in the N^−^ treatment but only very slightly (Figure S2), suggesting a limited control of genotype leaf [Si] on competition outcomes. However, plasticity in plant height and plasticity in leaf [Si] to the presence of a neighbour were positively related for both N treatments (Figure 4b) and within *intra‐* and *intergenotypic culture* (Figure S3), suggesting that increased leaf [Si] for plants in interaction implied an increase in plant height. In contrast, plasticity of above‐ground biomass to plant–plant interactions was not related to that of leaf [Si], except slightly in N^−^ (Figure 4b; Figure S3).

## 4 DISCUSSION

We demonstrate that durum wheat genotypes markedly differ in both their Si concentrations and response to N fertilisation and plant–plant interactions. Despite contrasting responses among genotypes, N fertilisation predominantly decreased leaf Si concentrations. The responses to plant–plant interactions were less clear, with both increases and decreases in leaf [Si] in the presence of a neighbour among the studied wheat genotypes. The genotypic responses to plant–plant interactions were rather consistent between *intra‐* and *intergenotypic cultures*, even though neighbour identity seemed to play a slight role in Si concentrations, at least in N^−^. We also show that the leaf [Si] of a given genotype has a limited influence on its biomass gain/loss when mixed with a neighbour. However, we show that increased leaf [Si] in response to competition was associated with increased plant height, which could have a role in light capture.

The strong decrease in Si concentrations following N fertilisation confirms our hypothesis and previous studies using natural grassland/pasture species (Johnson et al., 2021; Massey et al., 2007; Minden et al., 2021; Quigley et al., 2020). The results are also in line with the resource availability hypothesis, which proposes higher levels of defence in resource‐limited environments (Coley et al., 1985; Endara & Coley, 2011). Since Si is thought to incur lower C costs than C‐based structural/defensive compounds (Raven, 1983), this might also reflect a selective advantage of plants reducing leaf construction/defence costs when resources are limiting (Minden et al., 2021), but the underlying mechanism remains unclear (Hodson & Guppy, 2022). Although N deficiency might directly increase the expression of Si transporters (Wu et al., 2017), our results suggest a N‐driven ‘dilution effect’ on leaf [Si] (Hodson & Guppy, 2022; Jarrell & Beverly, 1981) since we found a strong negative relation between biomass and leaf [Si]. This likely explains the strong negative relationship between the plasticity of biomass and that of leaf [Si] to N fertilisation. The significant interactions between wheat Si concentrations, total above‐ground biomass and responses to N fertilisation stress the need to combine data on total Si content and total dry matter content, wherever possible (Jarrell & Beverly, 1981).

Despite the significantly lower biomass of plants in mixtures compared with that of plants grown alone in pots, growth modality did not significantly influence leaf [Si] overall. However, the response of leaf [Si] to a neighbour presence strongly varied among the 19 wheat genotypes, and neighbour identity influenced the responses of leaf [Si] to plant–plant interactions in N^−^, in the *intergenotypic culture* treatment. So far, facilitation for Si uptake in the rhizosphere has been demonstrated at the interspecific level with functionally contrasting species (e.g. contrast in Si demand and/or nutrient‐acquisition strategies; Ning et al., 2021). Our results suggest that both competition and facilitation for Si uptake might exist at the intraspecific level with durum wheat genotypes. Our comprehension of root‐related processes influencing Si mobilisation in the rhizosphere is still limited, despite some progress in recent years (de Tombeur, Cornelis, et al., 2021; Frew et al., 2017; Gattullo et al., 2016). Grasses release siderophores (i.e. low‐molecular weight chelators) in the soil solution to acquire limited nutrients (Ma, 2005; Oburger et al., 2014; Römheld, 1991), which also increase Si availability (Gattullo et al., 2016). This mechanism could possibly explain the increases of leaf [Si] of some mixtures (either *intra‐* and *intergenotypic* mixtures), and why some genotypes (especially GQ4X76) consistently induced an increase of leaf [Si] of their neighbours.

A potential impact of genotype leaf [Si] on competition outcomes might be expected, since Si is involved in traits linked with plant architecture and light capture (Ando et al., 2002; de Tombeur, Cooke, et al., 2021; Yamamoto et al., 2012; Zanão Júnior et al., 2010). However, despite a slightly positive relationship between genotype‐mean leaf [Si] in *single* and the response of above‐ground biomass to competition in the N^−^ treatment, genotype leaf [Si] did not appear to play a major role in intra‐ or intergenotypic competition outcomes. Nevertheless, increased leaf [Si] in response to competition was associated with increased plant height, and this was the case for both N and mixture treatments. Si might play an indirect role in intraspecific competition through its influence on plant height, given that this trait is often associated with a strong competitive ability in wheat (Thomas et al., 1993; Yenish & Young, 2004) and more generally with light capture (Falster & Westoby, 2003; Violle et al., 2009). Height gain following Si fertilisation is, however, also associated with straighter leaves with lower leaf insertion angle (Zanão Júnior et al., 2010), which might in turn reduce the light extinction coefficient inside the canopy (Ando et al., 2002). In any case, this finding opens up new research directions on Si and plant–plant interactions in both natural and agroecosystems which remain strikingly scarce to date (but see Garbuzov et al., 2011; Ning et al., 2017, 2021).

Several perspectives arise from the results discussed above. First, the observed intragenotypic variation in leaf [Si] might be linked to the expression of Si transporters (Ma et al., 2007), which should be tested among the 180 EPO durum wheat genotypes. Finding a consistent pattern among genotype leaf [Si] and the expression of Si transporters at the intraspecific level would improve our understanding of the evolutionary path of Si uptake by vascular plants (Deshmukh et al., 2020; Deshmukh & Bélanger, 2016). Second, since genotype leaf [Si] directly influences levels of silica‐based defences (Hartley et al., 2015; McLarnon et al., 2017) and their responses to abiotic stresses (Thorne et al., 2022), breeding for Si‐rich crop genotypes may have benefits for reducing pesticide inputs, especially in low‐nutrient and/or herbivore susceptible areas (Christian et al., 2022). Regarding N fertilisation, genotypes for which leaf [Si] did not decrease might be retained by plant breeders to limit the N‐driven decrease in silica‐based defences. Regarding plant–plant interactions, genotypes for which leaf [Si] increased when mixed with a neighbour might be preferred for their potentially greater ability to accumulate Si and cope with environmental stresses, either in intra‐ or intergenotypic cultures. Third, the strong N‐driven decrease in plant Si concentrations—and most likely resulting silica‐based defences—may have detrimental effects on herbivore attacks (Johnson et al., 2021) and resulting crop sustainability and food security (Sundström et al., 2014). Such negative feedback could be mitigated through the use of Si fertilisers, even though it comes with potential drawbacks and significant C footprints (Thorne et al., 2020). Implementing agricultural practices that have positive impacts on soil–plant Si mobility (e.g. cereal‐legume intercropping, cover crops; no‐till farming; de Tombeur, Roux, et al., 2021; Li et al., 2020) might mitigate this negative feedback. Finally, our results suggest the existence of ‘good neighbours’ that facilitate Si uptake. Future research should identify which root chemical and physical traits characterise these facilitators, for the future development of productive and stress‐resistant genotypes mixtures, that is, ideomixes (Litrico & Violle, 2015). Furthermore, facilitation/competition for plant Si uptake should be tested for different soil types with contrasting Si availability because the effect of plant–plant interactions on plant Si uptake is influenced by soil Si availability (Ning et al., 2021).

## AUTHOR CONTRIBUTIONS

Cyrille Violle, Florian Fort and Taïna Lemoine conceived the ideas; Cyrille Violle, Florian Fort, Hélène Fréville and Taïna Lemoine designed the experiment; Taïna Lemoine and Sarah J. Thorne collected the data; Felix de Tombeur analysed the data and led the writing of the manuscript. All authors contributed critically to drafts and gave final approval for publication.

## CONFLICT OF INTEREST

C.V. is an Associate Editor of Functional Ecology but took no part in the peer review and decision‐making processes for this paper. The authors declare that they have no competing interests.

## Supporting information


Appendix S1

## Data Availability

Data are available via the Dryad Digital Repository https://doi.org/10.5061/dryad.x3ffbg7nj (de Tombeur et al., 2022).
