# Supplementary material for: Nitrogen availability and plant–plant interactions drive leaf silicon concentration in wheat genotypes
Source: Funct Ecol. 2022 Sep 1;36(11):2833–44. doi: 10.1111/1365-2435.14170 (PMC9804457; doi:10.1111/1365-2435.14170)
Supplement: Supplementary file 1 — Appendix S1 [file FEC-36-2833-s001.docx]

**Supporting Information**

**Nitrogen availability and plant-plant interactions drive leaf silicon concentration in wheat genotypes**

*Functional Ecology*

Felix de Tombeur^1,2*^, Taïna Lemoine^1,3^, Cyrille Violle^1^, Hélène Fréville^3^, Sarah J. Thorne^4^, Sue E. Hartley^5^, Hans Lambers^2^, Florian Fort^6^

^1^CEFE, Univ Montpellier, CNRS, EPHE, IRD, Montpellier, France

^2^School of Biological Sciences and Institute of Agriculture, The University of Western Australia, Perth, Australia

^3^AGAP, Univ Montpellier, CIRAD, INRAE, Institut Agro, Montpellier, France

^4^Department of Biology, University of York, York, United Kingdom

^5^School of Biosciences, University of Sheffield, Sheffield, United Kingdom

^6^CEFE, Univ. Montpellier, L’Institut agro, CNRS, EPHE, IRD, Montpellier, France

*Corresponding author ; felix. detombeur@cefe.cnrs.fr

**
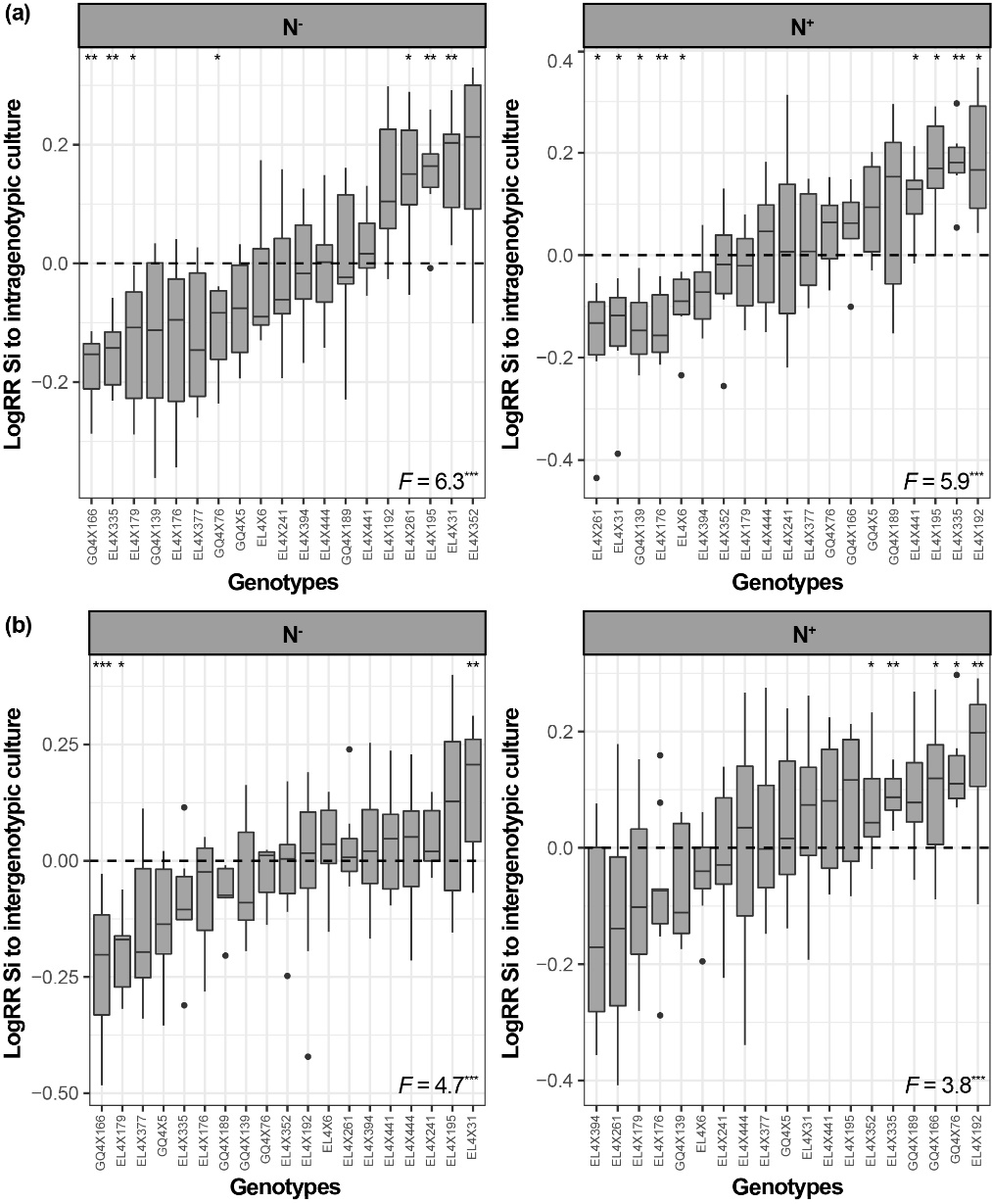
**

**Fig. S1 –** Variation in log response ratios (logRR) of leaf silicon (Si) concentrations to *intragenotypic culture* in (a) and to *intergenotypic culture* in (b) for both nitrogen (N) treatments. Data are ranked by increasing genotype-mean logRR. The central horizontal bar in each box shows the median, the box represents the interquartile range (IQR), the whiskers show the location of the most extreme data points that are still within a factor of 1.5 of the upper or lower quartiles, and black points are values that fall outside the whiskers. Results of ANOVA (*F*-values) conducted between the genotypes are given. LogRR significantly different from zero following Student *t-*tests are indicated with stars. ****p* < 0.001; ***p* < 0.01; **p* < 0.05; ns, not significant.


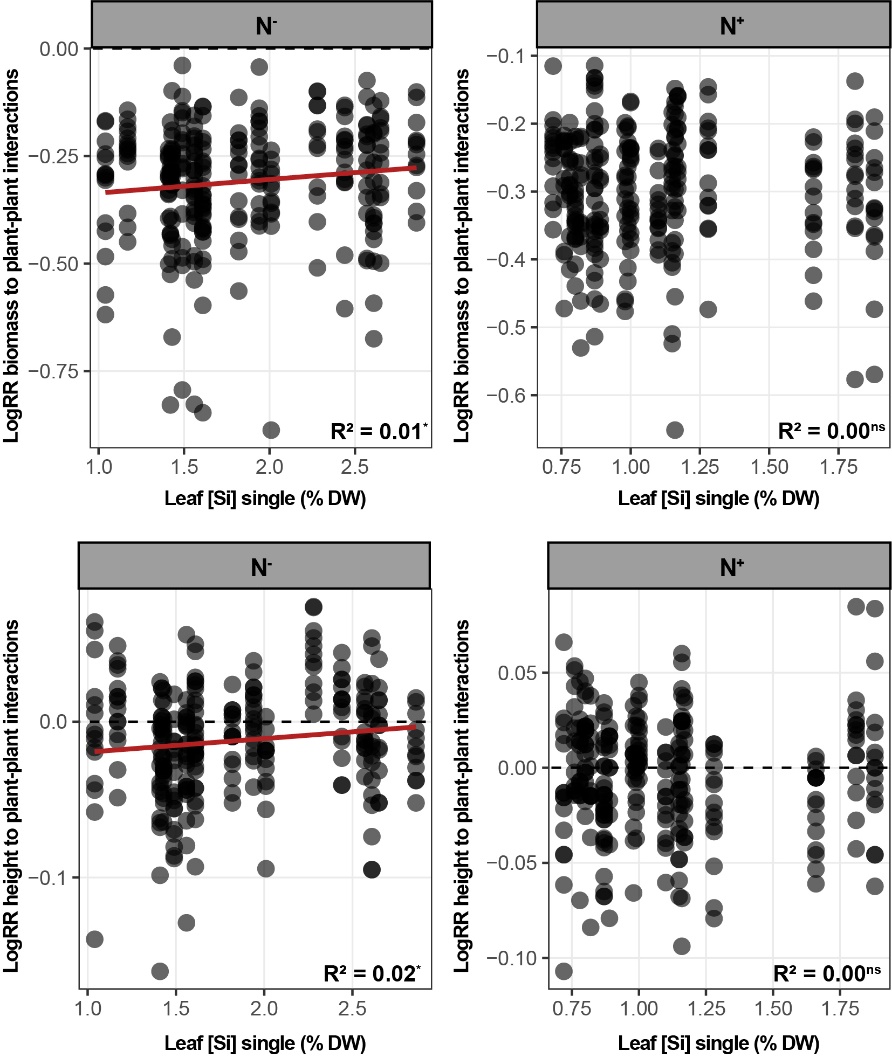


**Fig. S2 –** Relationships between genotype-mean leaf silicon concentrations ([Si]) in *single* and the log response ratio (logRR) of plant height and biomass to plant-plant interactions for both nitrogen (N) treatments. Both *intra-* and *intergenotypic culture* were considered together as ‘plant-plant interactions’ in the analyses (see Fig. S4 for separate analyses). Red lines indicate regression lines between variables, and multiple R-squared are given. ****p* < 0.001; ***p* < 0.01; **p* < 0.05; ns, not significant.


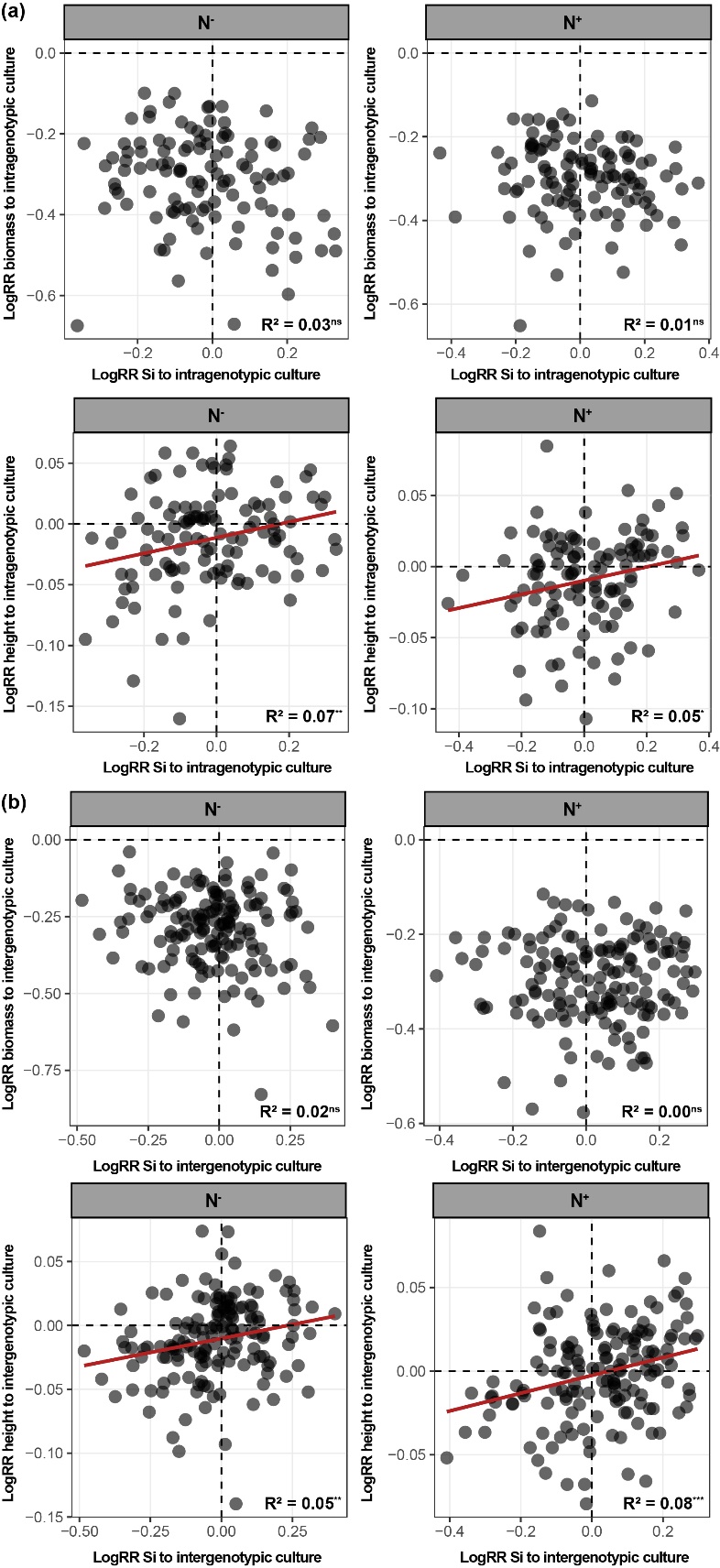


**Fig. S3 –** Relationships between the log response ratio (logRR) of leaf silicon (Si) concentrations and those of biomass and height to plant-plant interactions for both nitrogen (N) treatments, for the *intragenotypic culture* in (a) and for the *intergenotypic culture* in (b). Red lines indicate regression lines between variables, and multiple R-squared are given. ****p* < 0.001; ***p* < 0.01; **p* < 0.05; ns, not significant.

**
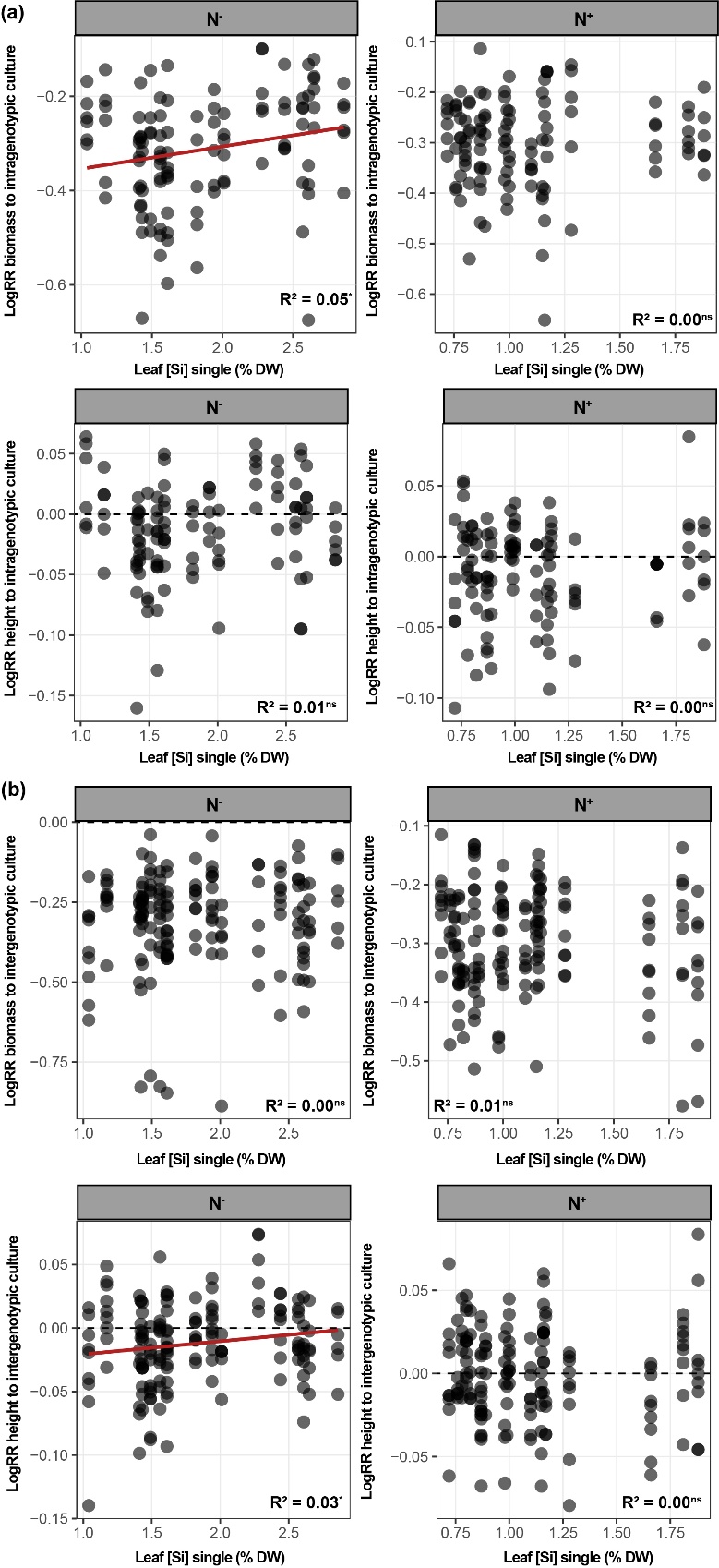
**

**Fig. S4 –** Relationships between genotype-mean leaf silicon concentrations ([Si]) and the log response ratio (logRR) of plant height and biomass to *intragenotypic culture* in (a) and to *intergenotypic culture* in (b), for both nitrogen (N) treatments. Red lines indicate regression lines between variables, and multiple R-squared are given. ****p* < 0.001; ***p* < 0.01; **p* < 0.05; ns, not significant.

**Table S1** – Results of the mixed-effect models (genotype as random factor) testing the effects of leaf silicon (Si) concentrations on plant aboveground biomass and height for the treatments *intra-* and *intergenotypic culture*, for both nitrogen (N) treatments.

|  | **Intragenotypic culture** | | | | | | **Intergenotypic culture** | | | | | |
| --- | --- | --- | --- | --- | --- | --- | --- | --- | --- | --- | --- | --- |
|  | N^-^ | | | N^+^ | | | N^-^ | | | N^+^ | | |
|  | Slope | F-value | p-value | Slope | F-value | p-value | Slope | F-value | p-value | Slope | F-value | p-value |
| Biomass ~ Leaf Si | -0.2 | 0.3 | 0.56 | -1.0 | 0.8 | 0.37 | -0.6 | 3.1 | 0.08 | -0.5 | 0.4 | 0.52 |
| Height ~ Leaf Si | 3.6 | 13.1 | <0.001 | 6.4 | 16.6 | <0.001 | 1.3 | 4.2 | <0.05 | 4.3 | 13.1 | <0.001 |
